# The Health of London

**Published:** 1901-12-28

**Authors:** 


### THE HEALTH OF LONDON.

The report of the Medical Officer of Health of the County of London for the year 1900, which has just been issued, is as usual full of information regarding the health conditions of the metropolis. It appears that the increase in the population of London by no means continues to take place at the same rate as it used to do some years ago, having in fact declined from 21·3 per cent. in the period 1841–51 to 7·3 per cent. in the period 1891–1901. The natural increase in the London population in the period 1891-1900 (*i.e.*, the excess of births over deaths) was 490,518, while the actual increase, as shown by the census of 1901, was only 311,318. The loss of population by excess of emigration over immigration was, therefore, 179,200. Apparently the boundaries of the county are getting too small and London is overflowing into the surrounding districts. The marriage rate, which a few years ago showed signs of picking up a little after the depths to which it had fallen between 1887 and 1896, has gone down again, while the birth rate continues steadily to get less. The number of births registered in the County of London in 1900 was 131,362, giving a birth rate of 29·1 per 1,000 living, the lowest birth-rate recorded in London since civil registration began. The death-rate was lower than the year before, but not as low as in the years 1896–7–8. Out of 12 principal towns mentioned the death-rate of London was lower than any except Bristol, Bradford and West Ham. When, however, we compare the course of the London death-rate with that of foreign capitals it would seem that many of them are improving their condition in this respect at a much more rapid rate than is the case with us. Taking the whole period 1890–9 the London death-rate was lower than that of any of the ten other capitals specified, except Brussels, Amsterdam, Copenhagen, Stockholm, and Berlin; while in 1900 it exceeded the rates of all except Paris, St. Petersburg, Vienna, Rome, and New York. It is satisfactory to find that the infant mortality (per 1,000 births) was lower than in the preceding two years, still in certain districts it is a very unsatisfactory item, and one the extreme variation of which in different districts shows how immense is the influence of social and sanitary surroundings upon the chances of life of the infant population. When we find that the infant mortality in Hampstead is only 100 per 1,000 births, while in Holborn it is 240, and when we remember that these mortality returns take cognisance only of the cases in which death occurred (taking no heed of the possibly far more numerous cases in which diseases set up at that early period caused lifelong injury although they failed to kill within the year) we are able in some degree to estimate the serious influence upon the race which is exercised by the surroundings in which children are brought up. When we come to the subject of zymotic diseases we are met by the startling fact that with the exception of St. Petersburg, London had a higher death-rate from the six principal zymotic diseases than any of the ten foreign capitals with which its statistics are compared. On the other hand it had a lower zymotic death-rate than most of the large provincial towns. It seems clear then that we in England have but little right to exhibit self-complaisancy in regard to our control over infectious diseases. We are improving perhaps, but other nations are doing likewise—only more so.

